# Supplementary material for: Radiomics of Liver Metastases: A Systematic Review
Source: Cancers (Basel). 2020 Oct 7;12(10):2881. doi: 10.3390/cancers12102881 (PMC7600822; doi:10.3390/cancers12102881)
Supplement: Supplementary file 1 [file cancers-12-02881-s001.pdf]

Supplementary Material: Radiomics of Liver Metastases: A Systematic Review

Francesco Fiz, Luca Viganò, Nicolò Gennaro, Guido Costa, Ludovico la Bella, Alexandra Boichuk, Lara Cavinato, Martina Sollini, Letterio S. Politi, Arturo Chiti and Guido Torzilli

Table S1. QUADAS-2 evaluation of studies.

Study	Risk of Bias				Applicability Concerns		
	Patient Selection	Index Test	Reference Standard	Flow and Time	Patient Selection	Index Test	Reference Standard
Ahn SJ [37]	☹	☺	☺	☺	☺	☺	☹
Ahn SJ [36]	☺	☺	?	?	☺	?	?
Andersen IR [32]	☹	☹	☹	☺	☹	☹	☹
Beckers RCJ [38]	☺	☺	☺	☺	☺	☺	☺
Chatterjee A [57]	?	☺	?	?	?	☺	?
Cheng J [39]	☹	☺	☺	☺	☺	☺	☺
Dercle L [40]	?	☺	☹	☺	☺	☹	☹
Dercle L [35]	☺	?	?	?	☺	?	?
Dohan A [33]	☺	☺	☺	☹	☺	☹	☺
Gatos I [51]	?	☹	☹	☺	?	☹	☹
Jansen MJA [52]	☹	☹	☹	☺	☹	☹	☹
Klaassen R [41]	☺	☺	☺	☺	☺	☺	☺
Li Y [42]	☹	?	☺	☺	☺	?	☺
LI Z [53]	?	☺	☺	☺	☺	☺	☺
Liang HY [54]	☹	?	☺	☺	☺	☺	☺
Lubner MG [43]	☺	☺	☹	☺	☺	☺	☺
Martini I [44]	☺	☹	☹	☺	☺	☺	?
Meyer M [34]	☺	?	?	☹	☺	?	?
Peerlings [58]	☺	?	☺	☺	?	?	☺
Rahmim A [59]	☹	?	☹	☺	☹	☺	☺
Rao SX [45]	☹	☹	☺	☺	☹	☺	☺
Ravanelli M [46]	☺	☺	☺	☺	☺	☺	☺
Reimer RP [55]	?	☹	☺	☺	☹	☺	☹
Shur J [62]	☺	☺	☺	☺	☹	☺	☺
Simpson AL [47]	☹	☺	☹	☺	☺	☺	?
Song S [48]	?	☺	☺	☺	☺	☺	☺

Trebeschi [49]	☹	☹	☺	☺	☺	☹	☺
Van Helden EJ [61]	☺	☺	☺	☺	☺	☺	☺
Velichko YS [50]	☹	☺	?	☺	☹	☺	?
Wagner F [60]	☺	☺	☺	☺	☺	☺	☺
Weber M [63]	☺	☺	?	☺	☺	☺	☺
Zhang H [56]	☺	☹	☺	☺	☹	☺	☺

☺Low Risk ☹High Risk ? Unclear Risk

Table S2. Data about prediction of survival by radiomics in patients with liver metastases from colorectal cancer.

First Author	#	Imaging	Analyzed Imaging	Radiomic Features	Outcome	Data
Lubner MG [43]	77	CT	Pre-therapy	Entropy	OS	HR = 0.65, 95%CI = 0.44–0.95, p=0.03 at coarse filter level
Simpson AL [47]	198	CT	Pre-therapy	Tumor correlation and contrast	OS	HR = 2.35, 95%CI = 1.21–4.55, *p* = 0.013
				Future liver remnant energy and entropy	OS	HR = 2.15, 95%CI = 1.08–4.29, *p* = 0.029
					HDFS	HR=2.21, 95%CI=1.21–4.03, *p* = 0.010
Andersen IR [32]	27	CT	Pre/post therapy	Uniformity	OS	HR ranging from 1.5 × 10^{20} to 1.3 × 10^{49}, according to the filter used
				Entropy	OS	HR ranging from 0.16 to 0.63 according to the filter used
				Standard deviation	OS	HR ranging from 0.94 to 0.98, according to the filter used
Beckers RCJ [38]	70	CT	Pre-therapy	LM/parenchyma entropy ratio	OS	HR = 1.9, 95%CI = 0.95-3.78, *p* = 0.07
Dercle L [40]	667	CT	Pre/post therapy	Signature including Shape SI4, Log Z/X Entropy, GTDM Contrast	OS	HR = 44.3, 95%CI = 6.4-307.7, *p* < 0.001 for patients with high imaging quality; HR = 6.5, 95%CI = 1.8-23.6, *p* = 0.005 for patients with standard imaging quality
Dohan A [33]	230	CT	Pre/post therapy	SPECTRA score (cut-off 0.02)	OS	HR = 2.82, 95%CI = 1.85-4.28, median survival 1.210 years, 95%CI 1.035–1.385 vs. 2.497 years, 95%CI = 1.786 to 3.208, *p* <0.0001 in the training dataset; HR = 2.07, 95%CI = 1.34-3.20, median survival 1.418 years, 95%CI 1.181–1.656 vs 2.289 years, 95%CI 1·698–2·880, *p* < 0.0008 in the validation dataset
Rahmim A [59]	52	FDG PET	Pre-therapy	Heterogeneity (included into a predictive model)	OS	HR = 4.29, 95%CI = 2.15–8.57, *p* < 0.001
				Histogram uniformity (included into a predictive model)	EFS	HR = 3.20, 95%CI 1.73–5.94, *p* < 0.001
Ravanelli M [46]	43	CT	Pre/post therapy	Uniformity (cut-off ≥0.42) in the EGFR group	OS	RR = 6.94; 95%CI = 1.79–26.79, *p* = 0.005
					PFS	RR = 5.05, 95%CI = 1.74–14.66, *p* = 0.004
				Density (cut-off <53 HU) in the EGFR group	OS	RR = 3.7, 95%CI = 1.16–11.76, *p* = 0.028
Shur J [62]	102	CT; MRI	Pre-surgery	Minimum pixel value	PFS	HR = 1.66, 95%CI = 1.28–2.16, *p* < 0.001
				GLSZM small area emphasis	PFS	HR = 0.62, 95%CI = 0.47–0.83, *p* = 0.001
Van Helden EJ [61]	47	FDG PET	Pre-therapy	AUC-ISH	OS	HR = 0.77, 95%CI = 0.66–0.89, *p* < 0.01
					PFS	HR = 0.86, 95%CI = 0.76–0.97, *p* = 0.02

OS: overall survival, HDFS: hepatic disease-free survival, EFS: event-free survival, PFS: progression-free survival, GLSZM: gray level size zone matrix, GTDM: gray tone difference matrix, AUC-ISH: area-under-the-curve of cumulative SUV/Volume histograms, HR: hazard ratio, 95%CI: 95% confidence intervals; RR: relative risk.

Table S3. Data about prediction of response to chemotherapy by radiomics in patients with liver metastases from colorectal cancer.

First Author	#	Imaging	Analyzed Imaging	Radiomic Feature	Outcome	Data
Ahn SJ [36]	235	CT	Pre-therapy	Skewness on 2D	RECIST	0.02 ± 0.32 in responders vs. 0.33 ± 0.44 in non-responders, $p = 0.001$
				Mean attenuation in 3D	RECIST	82.94 ± 16.55 in responders vs. 71.76 ± 16.71 in non-responders, $p < 0.001$
				Standard deviation on 3D	RECIST	21.69 ± 6.99 in responders vs. 25.06 ± 6.39 in non-responders, $p = 0.001$
Beckers RCJ [38]	56	CT	Pre-therapy	Entropy	RECIST	6.65±0.26 in responders vs. 6.51±0.34 in non-responders, $p = 0.08$
Dercle L [40]	667	CT	Pre/post therapy	Signature including Shape SI4, Log Z/X Entropy, GTDM Contrast	RECIST	AUC = 0.80, 95%CI = 0.69–0.94 for patients with high imaging quality AUC = 0.72, 95%CI = 0.59–0.83 for patients with standard imaging quality
Liang HY [54]	53	MRI	Pre-therapy	Mean ADC values (cut-off 123.8)	RECIST	AUC = 0.79, 95%CI = 0.66–0.89, $p = 0.001$ ADC value104.3 ± 30.5 in responders vs. 150.1 ± 46.1 in non-responders
Rao SX [45]	21	CT	Pre/post therapy	Entropy variation after treatment	TRG	−5.13 in responders vs. +1.27 in non-responders, OR = 1.34, 95%CI=0.92–1.93
				Uniformity variation after treatment	TRG	+30.84 in responders vs. −0.44 in non-responders, OR = 0.95, 95%CI = 0.89–1.01
Ravanelli M. [46]	43	CT	Pre/post therapy	Uniformity (cut-off ≥0.42) in EGFR patients	RECIST	OR = 20, 95%CI = 1.85–217.4, $p = 0.01$
Van Helden EJ [61]	47	FDG PET	Pre-therapy	Entropy	RECIST	AUC = 0.74, 95%CI = 0.52–0.97
Zhang H [56]	26	MRI	Pre-therapy	Variance	Size change	446.07 ± 329.60 in responders vs. 210.23 ± 183.39 in non-responders, $p <$ 0.001, AUC = 0.729 95%CI = 0.661–0.790;
				Angular second moment	Size change	0.96 ± 0.02 in responders vs. 0.98 + 0.01 in non-responders $p < 0.001$, AUC = 0.773, 95%CI = 0.707–0.830

Andersen IR et al. study [32] and Dohan A [33] have a complex results presentation that cannot be summarized in this table. Please refer to the original papers for details. RECIST: response evaluation criteria in solid tumors, TRG: tumor regression grade, GTDM: gray tone difference matrix, ADC: apparent diffusion coefficient, AUC: area under the curve, OR: odds ratio, 95%CI: 95% confidence intervals.

Table S4. PRISMA checklist.

Section/Topic	#	Checklist Item	Reported on Page #
**Title**			
Title	1	Identify the report as a systematic review, meta-analysis, or both.	Title page
**Abstract**			
Structured summary	2	Provide a structured summary including, as applicable: background; objectives; data sources; study eligibility criteria, participants, and interventions; study appraisal and synthesis methods; results; limitations; conclusions and implications of key findings; systematic review registration number.	Title page
**Introduction**			
Rationale	3	Describe the rationale for the review in the context of what is already known.	2
Objectives	4	Provide an explicit statement of questions being addressed with reference to participants, interventions, comparisons, outcomes, and study design (PICOS).	N/A
**Methods**			
Protocol and registration	5	Indicate if a review protocol exists, if and where it can be accessed (e.g., Web address), and, if available, provide registration information including registration number.	18
Eligibility criteria	6	Specify study characteristics (e.g., PICOS, length of follow-up) and report characteristics (e.g., years considered, language, publication status) used as criteria for eligibility, giving rationale.	N/A
Information sources	7	Describe all information sources (e.g., databases with dates of coverage, contact with study authors to identify additional studies) in the search and date last searched.	Title page, 18
Search	8	Present full electronic search strategy for at least one database, including any limits used, such that it could be repeated.	18
Study selection	9	State the process for selecting studies (i.e., screening, eligibility, included in systematic review, and, if applicable, included in the meta-analysis).	18
Data collection process	10	Describe method of data extraction from reports (e.g., piloted forms, independently, in duplicate) and any processes for obtaining and confirming data from investigators.	18
Data items	11	List and define all variables for which data were sought (e.g., PICOS, funding sources) and any assumptions and simplifications made.	18
Risk of bias in individual studies	12	Describe methods used for assessing risk of bias of individual studies (including specification of whether this was done at the study or outcome level), and how this information is to be used in any data synthesis.	18
Summary measures	13	State the principal summary measures (e.g., risk ratio, difference in means).	18-19
Synthesis of results	14	Describe the methods of handling data and combining results of studies, if done, including measures of consistency (e.g., I^2) for each meta-analysis.	N/A
Risk of bias across studies	15	Specify any assessment of risk of bias that may affect the cumulative evidence (e.g., publication bias, selective reporting within studies).	5-6; Supplementary Table 1
Additional analyses	16	Describe methods of additional analyses (e.g., sensitivity or subgroup analyses, meta-regression), if done, indicating which were pre-specified.	N/A

Table S4. *Cont.*

**Results**			
Study selection	17	Give numbers of studies screened, assessed for eligibility, and included in the review, with reasons for exclusions at each stage, ideally with a flow diagram.	3
Study characteristics	18	For each study, present characteristics for which data were extracted (e.g., study size, PICOS, follow-up period) and provide the citations.	N/A
Risk of bias within studies	19	Present data on risk of bias of each study and, if available, any outcome level assessment (see item 12).	5-6, Supplementary Table 1
Results of individual studies	20	For all outcomes considered (benefits or harms), present, for each study: (a) simple summary data for each intervention group (b) effect estimates and confidence intervals, ideally with a forest plot.	N/A
Synthesis of results	21	Present results of each meta-analysis done, including confidence intervals and measures of consistency.	N/A
Risk of bias across studies	22	Present results of any assessment of risk of bias across studies (see Item 15).	5-6, Supplementary Table 1
Additional analysis	23	Give results of additional analyses, if done (e.g., sensitivity or subgroup analyses, meta-regression [see Item 16]).	N/A
**Discussion**			
Summary of evidence	24	Summarize the main findings including the strength of evidence for each main outcome; consider their relevance to key groups (e.g., healthcare providers, users, and policy makers).	16
Limitations	25	Discuss limitations at study and outcome level (e.g., risk of bias), and at review-level (e.g., incomplete retrieval of identified research, reporting bias).	16-17
Conclusions	26	Provide a general interpretation of the results in the context of other evidence, and implications for future research.	19
**Funding**			
Funding	27	Describe sources of funding for the systematic review and other support (e.g., supply of data); role of funders for the systematic review.	20

From*:* Moher D, Liberati A, Tetzlaff J, Altman DG, The PRISMA Group (2009). Preferred Reporting Items for Systematic Reviews and Meta-Analyses: The PRISMA Statement. PLoS Med 6(7): e1000097. doi:10.1371/journal.pmed1000097. For more information, visit: www.prisma-statement.org.

© 2020 by the authors. Licensee MDPI, Basel, Switzerland. This article is an open access article distributed under the terms and conditions of the Creative Commons Attribution (CC BY) license (http://creativecommons.org/licenses/by/4.0/).
